# Phase II testing of sunitinib: the National Cancer Institute of Canada Clinical Trials Group IND Program Trials IND.182–185

**DOI:** 10.3747/co.2007.132

**Published:** 2007-08

**Authors:** R. Buckstein, R.M. Meyer, L. Seymour, J. Biagi, H. MacKay, S. Laurie, E. Eisenhauer

**Affiliations:** * Division of Hematology, Odette Cancer Center, Toronto, Ontario; † National Cancer Institute of Canada Clinical Trials Group, Queens University, Kingston, Ontario.; ‡ Cancer Centre of Southeastern Ontario, Kingston, Ontario.; § The Princess Margaret Hospital, Toronto, Ontario.; || Ottawa Regional Cancer Centre, Ottawa, Ontario

**Keywords:** Sunitinib, receptor tyrosine kinase inhibitor, ind, angiogenesis, ncic, phase ii

## Abstract

Sunitinib (SU11248) is an orally bioavailable inhibitor that affects the receptor tyrosine kinases involved in tumour proliferation and angiogenesis, including vascular endothelial growth factor (vegf) receptors 1, 2, 3, and platelet-derived growth factor receptors alpha (pdgfra) and beta (pdgfrb). Because angiogenesis is necessary for the growth and metastasis of solid tumours, and vegf is believed to have a pivotal role in that process, sunitinib treatment may have broad-spectrum clinical utility. In the present article, we discuss the biologic and clinical rationales that have recently led the Investigational New Drug Program of the National Cancer Institute of Canada Clinical Trials Group to initiate four phase ii trials testing this agent in the following four different tumour types: relapsed diffuse large cell lymphoma, malignant pleural mesothelioma, locally advanced or metastatic cervical cancer and recurrent epithelial ovarian, fallopian tube, or primary peritoneal carcinoma.

## 1. INTRODUCTION

Significant preclinical and clinical research in oncology is focused on targeting malignant angiogenesis, because angiogenesis is common to most tumours and may contribute to disease pathogenesis and propagation. Numerous agents are currently under development. One class of anti-angiogenic agents includes the multi-targeted receptor tyrosine kinase inhibitors. Agents within this class often inhibit more than one of the receptors that initiate the intracellular signalling that culminates in an angiogenic phenotype. Sunitinib is one such agent. In the present article, we discuss the biologic and clinical rationales that have recently led the Investigational New Drug (ind) Program of the National Cancer Institute of Canada Clinical Trials Group (ncic ctg) to initiate four phase ii trials testing this agent in four different tumour types.

Expression by tumours of vascular endothelial growth factor (vegf) has been associated clinically with disease prognosis in many different types of malignancies. This expression is increased by diverse stimuli, including proto-oncogene activation and hypoxia, with the hypoxic state frequently arising in solid tumours because of inadequate perfusion. In addition to its angiogenic role, vegf also profoundly increases the permeability of the vasculature, thereby potentially contributing to tumour progression, because a leaky tumour endothelium enhances nutrient and catabolite exchange and lowers barriers to tumour cell migration and extravasation during metastasis.

Two high-affinity receptors for vegf with associated tyrosine kinase activity have been identified on human vascular endothelium: vegfr1/flt1 and vegfr2/kinase insert domain-containing receptor. Increasing evidence implicates not only vegf receptor signalling, but also platelet-derived growth factor receptor (pdgfr) signalling in tumour angiogenesis. Recent preclinical evidence suggests that inhibition of pdgfr signalling augments the antitumour and anti-angiogenic effects of vegfr inhibitors 1. In addition, pdgfr signalling is implicated in the autocrine growth of tumour cells and in the recruitment and regulation of tumour fibroblasts.

## 2. SUNITINIB

Sunitinib (SU11248) it is an orally bioavailable inhibitor that affects the receptor tyrosine kinases involved in tumour proliferation and angiogenesis, including vegf receptors 1, 2, 3, and pdgfra and pdgfrb. The demonstrated activity of sunitinib in gastrointestinal stromal tumours (gist)^2^, acute myeloid leukemia (aml)^3^, and renal cell carcinoma (rcc) 4 may be at least in part mediated by its anti-angiogenic effects—although in some of these instances, effects on other tyrosine kinases may also play a role.

With chronic oral dosing, sunitinib is expected to inhibit pdgf- and vegf-driven angiogenesis and, as a consequence, to limit solid tumour growth. Because angiogenesis is necessary for the growth and metastasis of solid tumours, and vegf is believed to have a pivotal role in that process, sunitinib treatment may have broad-spectrum clinical utility 5,6. Sunitinib also exerts direct antitumour activity on cells that express target receptor tyrosine kinases associated with tumour cell proliferation, such as Kit, pdgfr, and Ret.

Phase i testing of sunitinib has demonstrated single-agent activity in patients with rcc, gist, non-gist sarcomas, non-small cell lung cancer, colorectal cancer, neuroendocrine tumours, melanoma, prostate cancer, and thyroid cancer. Phase i testing in patients with aml has been completed. Phase ii and iii testing have included single-agent trials in metastatic rcc, imatinib-resistant gist, metastatic breast cancer, non-small cell lung cancer, and carcinoid and islet cell neuroendocrine tumours. Results of some of these trials have led to approval of sunitinib for specific indications by the U.S. Food and Drug Administration. The most promising results have included a report of phase ii testing in patients with metastatic rcc: among 63 patients, 25 (40%) achieved a partial response (pr) as determined by the Response Evaluation Criteria in Solid Tumors (recist), and an additional 17 (27%) had stable disease (sd) for ≥ 3 months. In a multinational, randomized, double-blind, placebo-controlled phase iii trial that included more than 300 patients with imatinib-resistant gist, results of an interim analysis demonstrated a median time-to-progression of 27.3 weeks in the sunitinib arm as compared with 6.4 weeks in the placebo group (*p* < 0.001) 2. Based on these results, and in response to a solicitation for studies by the Cancer Therapy Evaluation Program of the U.S. National Cancer Institute, the ind Program of the ncic ctg has initiated separate phase ii trials testing sunitinib in patients with progressive diffuse large B cell lymphoma, malignant pleural mesothelioma, locally advanced or metastatic carcinoma of the cervix, and recurrent carcinoma of the ovary (including fallopian tube and primary peritoneal carcinoma).

## 3.RATIONALE FOR ANGIOGENESIS INHIBITORS

### 3.1 Diffuse Large B Cell Lymphoma

Anthracycline-based chemotherapy with cyclophosphamide, doxorubicin, vincristine, and prednisone (chop) remains the standard of care for most patients with aggressive-histology lymphoma 7. Although the addition of rituximab (chop-r) has improved survival in the primary treatment of diffuse large B cell lymphoma (dlbcl) 8, the management of relapsed and refractory lymphomas remains problematic. Autologous stem cell transplantation has a well-defined role in a limited number of patients who are of an appropriate age and who demonstrate chemosensitivity before transplantation 9. Unfortunately, most patients with aggressive-histology lymphomas who relapse after autologous transplantation succumb rapidly to their disease; median overall survival ranges from 3 months to 7.7 months 10,11. Traditional palliative chemotherapy is associated with short rates of progression-free and overall survival 12–14. Newer therapies may provide useful options for patients who are not eligible for, or who are unresponsive to, stem cell transplantation, given that no standard of care exists for these patients.

Preclinical and clinical data that are now available support the important role of tumour angiogenic growth factors and angiogenesis in the pathogenesis and prognosis of lymphoma 15,16; vegf and vegfr are present in lymphoma cells and angiogenesis-associated parameters are important prognosticators 17. Murine models of dlbcl xenografts respond to treatment with antibodies to vegfr1 and 2, supporting the presence of autocrine vegfr1– and paracrine vegfr2–mediated pathways in lymphomagenesis 18. Endostatin, an anti-angiogenic drug, induced tumour stabilization or regression (or both) after chemotherapy or anti-CD20 therapy in a nod/scid mouse model of human high-grade lymphoma 19. A trial of the vegf antibody, bevacizumab, in relapsed aggressive-histology lymphoma showed some modest activity (pr = 5% and sd = 20%), with a relationship between vegf and vegfr expression suggesting a possible autocrine pathway in some patients 20, which is consistent with preclinical models 18. These results have led to phase ii combination studies of bevacizumab with chop and chop-r and a phase iii study comparing chop-r with or without bevacizumab for newly diagnosed patients with dlbcl is in its initial stages.

Furthermore, independent research undertaken at some ncic ctg centres demonstrated a response rate of 37% and a sd rate of 20% in 32 heavily pretreated patients with relapsed and refractory aggressive non-Hodgkin lymphoma using anti-angiogenic “metronomic” oral chemotherapy (cyclophosphamide 50 mg daily) and high-dose celecoxib 800 mg daily. One third of these patients had disease progression after autologous transplantation 21. The median response duration was 8.5 months and 5 patients had responses lasting from 12 to 26 months or more. In this trial, circulating endothelial cells (cecs) and circulating endothelial cell progenitors (ceps) declined in patients responding to treatment. In the bevacizumab study referenced earlier 20, cecs and plasma vegf declined during therapy, but no relationship to response was described.

These data provide a rationale for studying other vegf- and vegfr-targeted agents in dlbcl. Evaluation of cecs and ceps would be logical correlative questions to be included in such testing.

### 3.2 Malignant Pleural Mesothelioma

Malignant pleural mesothelioma is an unusual malignancy. Approximately 400 new cases are diagnosed annually in Canada 22, and 2000–3000 new cases annually in the United States 23. The disease is commonly associated with earlier asbestos exposure. A preponderance of patients are not candidates for surgical therapy, because they present with locoregionally advanced disease or are not medically suitable for such therapy. These patients are treated with palliative intent. Cisplatin-based chemotherapy is the treatment of choice, but median survival is only 12 months. No data describing benefits of chemotherapy for patients with progressive disease following cisplatin-based therapy are available. Additionally, many patients are not medically suitable for platinum-based treatment, or they decline the option. Clearly, new agents are needed in this disease.

Recent reports in the literature have suggested a link between angiogenesis and prognosis in malignant mesothelioma. Mesothelioma tumours and cell lines have been found to express vegf ligands 24,25, which could contribute to tumour-associated angiogenesis and lymphangiogenesis. In addition to vegf ligands, mesothelioma tumours and cell lines have also been shown to express the receptors for vegf ligands 24–27, suggesting that mesothelioma tumour cells may respond to vegf in an autocrine and paracrine manner. Additionally, an autocrine growth stimulatory effect of pdgf via pdgfr may play a role in the disease pathogenesis and in the metastatic potential. Because angiogenesis is felt to be important in the pathogenesis of mesothelioma, there is a good rationale for the use of anti-angiogenesis agents in this cancer.

### 3.3 Locally Advanced or Metastatic Cervical Cancer

Carcinoma of the cervix is the second most common malignancy in women after breast cancer. With the introduction of Papanicolaou smear screening programs, the incidence and mortality have decreased, but approximately 200,000 women worldwide still die from their disease each year 28.

Based on the results of several phase iii studies, standard approaches for many patients include potentially curative treatment using primary radiotherapy and concurrent platinum-based chemotherapy. Long-term survival is approximately 85% for patients with T1 disease, 70% for those with T2 disease, and 40% for those with T3 disease 29. However, even with the best currently available treatment, a significant proportion of patients will experience recurrence and eventually die. Some patients present with extensive disease that is not amenable to curative locoregional therapy, and in others, complete disease eradication is never achieved. The median survival of patients with advanced disease is only 9 months 30.

A number of chemotherapeutic agents have shown activity in advanced and metastatic cervical cancer, including cisplatin 31,32, paclitaxel 33, ifosfamide 34, and topotecan 35. As compared with single–agent cisplatin, treatment with cisplatin-based combination chemotherapy improves survival, but with very modest gains and increased toxicity 30. Responses to second-line chemotherapy are infrequent. The poor outlook for these patients warrants the development of novel therapeutic strategies that exploit abnormal tumour biology as a means of improving patient outcome. Furthermore, with the adoption of platinum-based chemoradiation as a standard of care for locally advanced disease, the search for non-platinum based therapy is essential.

Overexpression of vegf in adenocarcinomas of the cervix suggests that vegf is involved in tumour angiogenesis in this histologic subtype 36. One study suggested that higher pretreatment levels of vegf and vegf-c in patients with squamous cell carcinomas of the cervix correlated with poor outcome 37. A phase ii trial of bevacizumab (GOG 227A) in cervix cancer is currently under way, and although the data have not undergone complete analysis, the study has met endpoints for the first phase of accrual, and the second phase has been opened. Human papilloma virus–associated cervical cancer–derived cell lines co-express c-Kit and its ligand stem cell factor. These molecules are important determinants of cell survival and cell–cell interaction. Other receptor tyrosine kinases have also been implicated in the development and progression of cervical carcinoma. Given the poor outlook for patients with advanced cervical cancer and the central role played by receptor tyrosine kinases in tumour proliferation and angiogenesis, it would therefore be rational to test a novel, multi-targeted inhibitor of angiogenesis in patients with unresectable, locally advanced or metastatic cervical carcinoma.

### 3.4 Recurrent Epithelial Ovarian, Fallopian Tube, and Primary Peritoneal Carcinoma

Ovarian cancer is the leading cause of gynecologic cancer deaths in North America. In 2005, an estimated 22,000 and 2400 women were diagnosed with this disease in the United States and Canada respectively; at least 70% would be expected to die of their disease 38.

Standard initial treatment includes debulking surgery followed by combination chemotherapy that includes a platinum drug and a taxane 39. Patients with primary peritoneal carcinomatosis and advanced fallopian tube cancers are generally managed in a comparable manner; thus, these patients are commonly included in clinical trials evaluating therapies for ovarian cancer. Despite aggressive primary management, most patients with ovarian cancer will relapse and die of their disease. Systemic chemotherapy for relapsed or refractory disease can have important palliative effects, but treatment is not curative 40. An important determinant of survival in recurrent ovarian cancer is the platinum-free interval (pfi), which is the time from completion of initial platinum-based therapy to first evidence of recurrence 41. Patients with a pfi of at least 6 months have a higher likelihood of response to second therapies and superior survival 42. Importantly, clinicians and patients must balance chemotherapy toxicities with quality of life considerations when considering treatment for relapsed or refractory disease. Novel and more promising systemic treatment options are required.

Indicators of enhanced angiogenesis, such as circulating levels of vegf and tissue microvessel density, have been correlated with the presence of metastasis and survival in ovarian cancer 43. The vegf signalling pathway appears to contribute to growth and progression in 80% or more of all ovarian cancers 44, and pdgf and its receptor pathway have also been implicated in disease progression 45. Hence, targeted therapies against angiogenesis signalling pathways may interrupt malignant growth potential. This proof of principle has been established through observations of antitumour activity with the anti-vegf monoclonal antibody bevacizumab when used either as a single agent in recurrent disease or in combination with low-dose metronomic chemotherapy 46–48. On the basis of these initial clinical results, coupled with a strong biologic rationale, and based on an important need, priority must be given to an evaluation of the activity of alternative angiogenesis inhibitors for patients with ovarian cancer and related diseases.

## 4. NCIC CTG IND TRIALS TESTING SUNITINIB

Based on the strong biologic rationale linking angiogenesis with disease progression, and with the promising results of sunitinib in treating rcc, gist, and other cancer types, the ncic ctg, in conjunction with the Cancer Therapy Evaluation Program of the U.S. National Cancer Institute, has initiated four trials testing this agent in the management of relapsed dlbcl (ind.182); malignant pleural mesothelioma (ind.183); squamous cell, adenosquamous, or adenocarcinoma of the cervix (ind.184); and advanced or metastatic epithelial ovarian, fallopian tube carcinoma, or primary peritoneal carcinoma (ind.185). All are non-randomized, non-blinded, multicentre phase ii trials. Key objectives and inclusion and exclusion criteria shared by all four trials are summarized in the next few subsections, and Table I provides important study-specific details.

Among the common objectives are these:

- Assess the efficacy and toxicity of sunitinib.
- Document response rates, objective progression rates, and response durations.
- Assess dynamic biomarkers of angiogenesis in select studies and centres.

The primary endpoint of each study is to determine the overall response defined by International Workshop Criteria (for lymphoma) or recist (for cervical and ovarian cancers), and the modified recist criteria for mesothelioma 49. The design of each of the trials includes common features such as the inclusion and exclusion criteria (Table II).

### 4.1 Safety Issues, Including Drug Interactions

The most frequent adverse events seen following sunitinib treatment are constitutional (fatigue or asthenia), gastrointestinal (nausea, vomiting, diarrhea, abdominal pain, anorexia, stomatitis, dysgeusia), and hematologic (neutropenia, thrombocytopenia). Hand–foot syndrome and skin discoloration are also seen 4. Most of these adverse events are grades 1 and 2, but at higher doses (75 mg), grades 3 and 4 fatigue or asthenia were dose-limiting but readily reversible on discontinuation of treatment 50. Tumour-related hemorrhage can occur with sunitinib, and in the case of pulmonary tumours, it may present as severe and life-threatening hemoptysis or pulmonary hemorrhage.

Sunitinib may induce asymptomatic mild declines in left ventricular ejection fraction (lvef) that are primarily reversible upon drug reduction or discontinuation. Infrequently (1.5%), grade 3 reductions in lvef have been seen in some trials, and symptomatic systolic dysfunction has been documented in 1% of patients. Because patients who presented with cardiac events within 12 months before sunitinib administration were excluded from clinical trials, whether patients with these concomitant conditions may be at higher risk of developing drug-related left ventricular dysfunction is unknown. Patients should be carefully monitored for signs of cardiac dysfunction, and baseline and periodic evaluations of lvef should be performed in patients with risk factors for cardiac disease such as prior anthracycline use or mediastinal radiation (product monograph). Sunitinib has been shown to prolong the QT interval in a dose-dependent manner, which may lead to an increased risk for ventricular arrhythmias such as torsade de pointes. Torsade de pointes has been observed in <0.1% of sunitinib-exposed patients. Sunitinib should be used with caution in patients with a history of arrhythmias, QT prolongation, pre-existing cardiac disease (product monograph). Hypertension is a common side effect of this class of angiogenesis inhibitors, and therefore strict guidelines for blood pressure monitoring and recommendations for antihypertensive medications and dose reductions by grade are indicated in the each of the four ind protocols. Each protocol also includes dose attenuation schedules if pre-specified toxicity criteria are observed.

Sunitinib malate is metabolized primarily by liver enzymes, particularly CYP3A4. Thus, CYP3A4 inducers—for example, rifampin, dexamethasone—and CYP3A4 inhibitors—for example—grapefruit juice, ketoconazole—should be avoided, and certain potent inhibitors and inducers are contraindicated. Based on clinical symptoms, dose reductions of the CYP3A4 inhibitors are recommended. Concomitant treatment with dysrhythmic drugs—that is, terfenadine, quini-dine, procainamide, disopyramide, sotalol, probucol, bepridil, haloperidol, risperidone, indapamide, and flecainide—is not recommended.

### 4.2 Assessment of Anti-angiogenic Activities

There is a strong rationale for evaluating circulating endothelial cells (cecs) and circulating endothelial cell progenitors (ceps) as biomarkers of angiogenesis, because the development of biomarkers that describe the angiogenic profile of a tumour type and might predict for response to anti-angiogenic agents carries great potential 51,52. Resting and activated endothelial cells are increased in the peripheral blood of cancer patients 53 and may serve as more reliable surrogate markers of angiogenesis than do soluble circulating angiogenic growth factors and microvessel density 54. In preclinical models, cecs and ceps s have been found to be increased in lymphoma 55, in the blood of breast cancer and lymphoma patients 53,56, and in patients with myelodysplastic syndromes 57. Recently, a number of investigators demonstrated that ceps (measured by four-colour flow cytometry) can serve as biomarkers for angiogenic responsiveness to vegfr2 blocking antibody or a thrombospondin mimetic peptide in several mouse strains 58. In addition, other preclinical studies have shown that other anti-angiogenic agents, such as Endostatin (EntreMed Rockville, MD, USA), caused reduced levels of cecs and their cep subset 59. Based on this background, and the ability to capitalize on Canadian strengths in this line of investigation through collaboration with Shaked and colleagues 60, serial analysis of cecs and ceps will be included for selected patients in ind.182 testing of sunitinib in patients with dlbcl to assess whether these biomarkers are predictive of response.

## 5. SUMMARY AND FUTURE DIRECTIONS

Sunitinib is an orally bioavailable inhibitor affecting receptor tyrosine kinases involved in tumour proliferation and angiogenesis. It has important antitumour activity in patients with metastatic rcc and in patients with gist who are resistant to imatinib. Given that neoangiogenesis is virtually ubiquitous in cancer and contributes to disease pathogenesis and propagation, it is logical to test sunitinib in the four cancers described earlier. If activity is demonstrated, future directions would include developing appropriate sunitinib-based combination regimens with chemotherapy or other targeted therapies for these malignancies. Insights gained from serial cec and cep measurements may help to identify patients likely to respond and may validate the anti-angiogenic mechanism of action of sunitinib.

## Figures and Tables

**TABLE I:** Study design for four prospective phase ii studies using sunitinib by the National Cancer Institute of Canada Investigational New Drug (ind) program

Study	Design	Sample size	Specific inclusion criteria	Dosing	Treatment duration
ind 182	Dual-stage design with 15 patients in stage 1 and 10 patients in stage 2 Drug active if ≥3 responses	Up to 25	Relapsed or refractory DLBCL [includes thymic (mediastinal)] One to two prior chemotherapy regimens (one of which must have been doxorubicin-based) and may have received one other non-chemotherapy regimen such as radiation May be relapsed post ASCT lvef by muga > lln	37.5 mg PO daily for 4-week cycles continuously	Up to 12 months in patients achieving cr, pr, or sd
ind 183	Two-stage design— Cohort 1: 16 patients in stage 1 and 10 patients in stage 2 Drug active if ≥5 responses Cohort 2: 17 patients in stage 1 and 15 patients in stage 2 Drug active if ≥8 responses	Cohort 1: Up to 26 Cohort 2: up to 32	Malignant pleural mesothelioma Advanced or metastatic disease Two cohorts— cohort 1: previously treated, one prior cisplatin-containing regimen permitted, previous radiation and egfr inhibitors cohort 2: previously untreated	50 mg po daily for 4 weeks of 6-week cycles	Indefinite for patients achieving cr, pr or sd
IND 184	Two-stage design with 18 patients in stage 1 and 14 patients in stage 2 Drug active if ≥4 responses	Up to 32	Squamous cell carcinoma Adenosquamous carcinoma or adenocarcinoma of the cervix Unresected, locally advanced, or metastatic disease Neoadjuvant or adjuvant chemotherapy, concurrent chemoradiation permitted Up to one prior chemotherapy regimen for recurrent metastatic disease	50 mg po daily for 4 weeks of 6-week cycles	cr + 2 cycles Stable pr + 2 cycles sd: up to 6 cycles
ind 185	Two-stage design with 15 patients in stage 1 and 10 patients in stage 2 Drug active if ≥3 responses	Up to 25	Epithelial ovarian carcinoma, fallopian tube carcinoma, or primary peritoneal cancer Advanced or metastatic Minimum of one and maximum of two prior chemotherapy regimens (one must be cisplatinum-containing) Up to one prior hormonal therapy	50 mg po daily for 4 weeks of 6 week cycles	cr + 2 cycles Stable pr + 2 cycles sd: up to 6 cycles

dlbcl = diffuse large B cell lymphoma; po = orally; cr = complete response; pr = partial response; sd = stable disease; asct = autologous stem-cell transplant; lvef = left ventricular ejection fraction; muga = multiple gated acquisition scan; lln = lower limit of normal; egfr = epidermal growth factor receptor.

**TABLE II:** Common eligibility criteria for four Investigational New Drug (ind) program trials testing sunitinib by the National Cancer Institute of Canada Clinical Trials Group

Common inclusion criteria Life expectancy of 12 weeks Presence of measurable disease Duration of 28 days since prior treatment Eastern Cooperative Oncology Group Performance Status 0–1 (up to 2 in some protocols) Ability to take oral medications Non-use or ability to discontinue selected CYP3A4 inhibitors or inducers
Common exclusion criteria Prior therapy with anti-angiogenic or multi-targeted tyrosine kinase inhibitors Presence of brain metastases Use of concurrent anticancer therapy Presence of uncontrolled hypertension Presence of serious medical conditions or cardiac disease Use of warfarin in therapeutic doses Occurrence of stroke, transient ischemic attack, or pulmonary embolism within 12 months Use of drugs with cardiac proarrhythmic potential Pregnancy or lactation Known positivity for the human immunodeficiency virus
